# Transcriptome analysis of chicken kidney tissues following coronavirus avian infectious bronchitis virus infection

**DOI:** 10.1186/1471-2164-14-743

**Published:** 2013-10-30

**Authors:** Feng Cong, Xiaoli Liu, Zongxi Han, Yuhao Shao, Xiangang Kong, Shengwang Liu

**Affiliations:** 1 Division of Avian Infectious Diseases, State Key Laboratory of Veterinary Biotechnology, Harbin Veterinary Research Institute, The Chinese Academy of Agricultural Sciences, Harbin 150001, The People’s Republic of China

**Keywords:** Infectious bronchitis virus, Kidney, Microarray, Transcriptome

## Abstract

**Background:**

Infectious bronchitis virus (IBV), a prototype of the *Coronaviridae* family, is an economically important causative agent of infectious bronchitis in chickens and causes an acute and highly contagious upper respiratory tract infections that may lead to nephritis. However, the molecular antiviral mechanisms of chickens to IBV infection remain poorly understood. In this study, we conducted global gene expression profiling of chicken kidney tissue after nephropathogenic IBV infection to better understand the interactions between host and virus.

**Results:**

IBV infection contributed to differential expression of 1777 genes, of which 876 were up-regulated and 901 down-regulated in the kidney compared to those of control chickens and 103 associated with immune and inflammatory responses may play important roles in the host defense response during IBV infection. Twelve of the altered immune-related genes were confirmed by real-time RT-PCR. Gene ontology category, KEGG pathway, and gene interaction networks (STRING analysis) were analyzed to identify relationships among differentially expressed genes involved in signal transduction, cell adhesion, immune responses, apoptosis regulation, positive regulation of the I-kappaB kinase/NF-kappaB cascade and response to cytokine stimulus. Most of these genes were related and formed a large network, in which IL6, STAT1, MYD88, IRF1 and NFKB2 were key genes.

**Conclusions:**

Our results provided comprehensive knowledge regarding the host transcriptional response to IBV infection in chicken kidney tissues, thereby providing insight into IBV pathogenesis, particularly the involvement of innate immune pathway genes associated with IBV infection.

## Background

Avian infectious bronchitis virus (IBV) is a gamma coronavirus in the family *Coronaviridae*, which has been identified as the causative agent of infectious bronchitis (IB) as well as serious acute viral respiratory and urogenital diseases in commercial chickens flocks worldwide [1,2]. Infected chickens develop respiratory symptoms, kidney and oviduct lesions, reduced egg production with poor egg quality, and possible secondary complications [3,4]. IBV can replicate within the epithelial surfaces of the kidneys and cause granular degeneration, vacuolation, and desquamation of the tubular epithelium, and massive infiltration of heterophils in the interstitium. IBV-induced kidney lesions are typically characterized by interstitial nephritis and tubule lesions that are most prominent in the medulla [5,6].

Interactions between viruses and hosts occur at two levels: viral capacity to gain access to the target cell and competition between the viruses and host cells to control the cellular protein synthesis machinery. The virus/host interactions are largely determined by the virulence of the pathogen and the host immune response [7], and may lead to changes in host gene expression. However, many aspects of IBV-host interactions remain unclear. Elucidation of the basis of the interactions between IBV and kidney cells will provide new insights into the immune mechanisms underlying host antiviral strategies and the pathogenesis of viral infection. To date, a limited number of studies have examined host gene expression in response to IBV infection on a relatively large scale using microarrays or two-dimensional gel electrophoresis [8,9]. Gene expression alterations in avian embryonic tissues infected with IBV isolate B8358 were evaluated using a microarray containing 1191 unique chicken and turkey gene transcripts. Regulated expression has been established with several functional gene classes and pathways, including those coding kinases, interferon (IFN)-induced genes, chemokines, adhesins, vesicular trafficking and fusion proteins genes, extracellular matrix protein genes, the cell cycle, cell metabolism, physiology, and development, translation, RNA binding, lysosomal protein degradation and ubiquitination-related genes [10]. In addition, the gene transcription profiles of tracheal epithelial cells were examined 3 days postinfection with an attenuated IBV-Massachusetts strain [11]. These authors investigated 25 direct immune-related genes and found an up-regulation in toll-like receptor (TLR)2, TLR3, IFN-induced antiviral genes (Mx), and genes responsible for cytotoxic T cell killing, such as the Fas antigen and granzyme-A. More recently, changes in ex vivo and in ovo protein expression in Vero cells as well as chicken trachea and kidney tissues were reported after IBV infection [8,9,12,13]. However, there is limited information available regarding transcriptomics of host kidney cells in response to IBV infection. The current study was designed to compare the transcriptomes of kidney cells in IBV-infected chickens to those of control chickens. A subset of genes of interest identified by microarray analysis was validated by quantitative real-time reverse transcription polymerase chain reaction (qRT-PCR). The putative importance of some of these genes in IBV pathogenesis and immune response were also analyzed. The global gene expression profiling of chicken kidney tissue after nephropathogenic IBV infection will enable a better understanding of the pathogenesis of IBV infection and extend the knowledge of the nature of virus-host interactions.

## Methods

### Animals, viral infection and detection in kidney

One-day-old specific pathogen-free (SPF) chickens were obtained from the Laboratory Animal Center, Harbin Veterinary Research Institute, the Chinese Academy of Agricultural Sciences (Harbin, China). All animal experimental procedures were approved by the Ethical and Animal Welfare Committee of Heilongjiang Province, China.

Twenty 1-day-old SPF White Leghorn chicks were housed in separate isolators and divided into two groups of 10 chicks each. Each chick in group 1 was intranasally inoculated with 0.1 mL of 10^5.5^ median embryo infectious doses of strain ck/CH/LDL/091022 [14] at 15 days of age. Chicks in group 2 served as virus-free controls. Two birds in group 1 died at 5 and 6 days postinoculation (dpi), respectively, and fresh tissue samples of kidney from each dead bird were collected at the time of death. The kidneys from one bird in the control group were also collected at each time point corresponding to the bird deaths and then stored at-80°C until further processed. Serum samples were collected at 12 days dpi from the remaining group 1 and control birds and assayed in triplicate using a commercial total antibody enzyme-linked immunosorbent assay (IDEXX Laboratories, Inc., Westbrook, MA, USA) according to the manufacturer’s instructions. Serum-to-positive ratios were calculated as described previously [15]. Individual serum titers were expressed as absorption values at an optical density at 630 nm according to the manufacturer’s instructions. Real-time RT-PCR was used to quantify viral load in the kidneys of two dead IBV-infected chickens as described previously [16]. The remaining birds in both groups were killed humanely 15 dpi and kidney tissues were collected for virus recovery as described previously [15].

### RNA extraction, reverse transcription, RNA labeling, and cRNA hybridization

Total RNA was extracted from kidneys of uninfected birds or those that died from IBV infection using TRIzol reagent (Invitrogen, Carlsbad, CA, USA) and then purified using the QiagenRNeasy Mini Kit (Qiagen, Valencia, CA, USA) according to the manufacturer’s instructions. RNA was quantified and qualified using the Agilent 2100 Bioanalyzer (Agilent Technologies, Palo Alto, CA, USA). Briefly, 2 μg of total RNA were converted to cDNA, synthesized to Cy3-labelled cRNA, and amplified for one round using a commercial array service (Agilent Technologies, America). The Cy3-labeled cRNA probes were hybridized to a 4 × 44 K Agilent custom chicken oligo microarray (design ID: 017698). Scanning of the arrays was performed according to standard protocols using a G2505C Scanner (Agilent Technologies).

### Microarray analysis

Microarray data were subjected to bioinformatic analysis to identify statistically significant changes in gene expression between samples using GeneSpring GX 11.0 software (Agilent Technologies). Annotations for the microarray genes were downloaded from the National Center for Biotechnology Information (http://www.ncbi.nlm.nih.gov/), the Gene Ontology (GO) (http://www.geneontology.org/), and UniProt (http://www.uniprot.org/) databases. The GO category (http://www.geneontology.org) based on biological process and KEGG (http://www.genome.jp/kegg/) pathway analysis was determined for differentially expressed genes and a probability (*p*)-value < 0.05 and a false discovery rate (FDR) < 0.05 was used as a threshold. Gene interaction networks were analyzed using the STRING (http://string-db.org/) database of known and predicted protein interactions, which included direct (physical) and indirect (functional) associations.

### Real-time RT-PCR for confirmation

Twelve genes of interest were selected for confirmation with 18S serving as an endogenous control. Specific primers and probes were designed using Beacon Designer software 7.5 (Premier Biosoft International, Palo Alto, CA, USA). The primers and probes used for the RT-PCR assays are listed in Table 1. Total RNA was extracted using TRIzol Reagent (TaKaRa Biotech Co., Ltd., Dalian, China) according to the manufacturer’s instructions. One-step real-time RT-PCR reactions were performed using the One Step PrimeScript® RT-PCR kit (TaKaRa Biotech Co., Ltd.) on the Light-Cycler® 480 real-time PCR system (Roche Diagnostics, Basel, Switzerland) according to the following steps: reverse transcription at 42°C for 10 min, denaturation at 95°C for 10 s and 40 cycles at 95°C for 5 s, 55°C for 20 s, and 72°C for 10 s, followed by a cooling step at 40°C for 10 s. All samples were assayed in triplicate in each reaction. The data were analyzed using the standard curve method available with the LightCycler® 480 Software ver. 1.5.

**Table 1:** Primers and probes used for the RT-PCR assays

**Gene**	**GenBank ID**	**Forward primers (5′-3′)**	**Probes (5′-3′)**	**Reverse primers (5′-3′)**
STAT1	NM_001012914	AAGCAAACGTAATCTTCAGGATAAC	(FAM)-CAAGAAGACCCGATACACATGGCAA-(BHQ1)	TTTCTCTCCTCTTTCAGACAGTTG
ISG12-2	NM_001001296	TTCCACTATCCAGTCTATCTCAATG	(FAM)-ACCTGCTCCTGGACCGATGCTTCTT-(BHQ1)	GTGAATCTGTCTGTAAAGGATGAAC
SOCS3	NM_204600	ACTGCGCCCCAGGTGATG	(FAM)-CTCCCGGCAGCAGCACCCC-(BHQ1)	GGGAACTTGCTGTGGGTGAC
TNFAIP6	NM_001037837	GAGGCAGCGAGAAAAATAGGTTTC	(FAM)-CCATCCAGCCAGCAGCACACAC-(BHQ1)	GCTTTTACTATGGGGTAACCAACTC
IRG1	NM_001030821	TCCGAGATGTGGGCAAAGAC	(FAM)-TCGCTTGCTTCTCTGAATGACCACA-(BHQ1)	CCTACTCCAAGGGTATCCAGAATC
SPP1	NM_204535	CCAGAACAGCCGGACTTTC	(FAM)-TGACATTCCTAGCAAGAGCCAAGAG-(BHQ1)	TGGAATCATTGTCATCATCATCATC
18S	FM165414	GGTTGCAAAGCTGAAACTTAAAGG	(FAM)-ACTCCTGGTGGTGCCCTTCCGTCAA-(BHQ1)	TGAGGTTTCCCGTGTTGAGTC
IFIT5	XM_421662	AAAAGAAGGCAAATCATGAGTACC	(FAM)-AATTCCTTGAAGAACTCCCTGCTGC-(BHQ1)	TGATCCTCTATTGATTCTTCCAGAC
MX1	NM_204609	AGAAAGCAATAAGAAAAGCCCAAG	(FAM)-AATGCTACCATTAGTGCCAGCCAC-(BHQ1)	ACCAGATTTCAAGGGAAATTAGTTC
OASL	NM_205041	AGCACTGGTACAAGGAGATGTTG	(FAM)-CTGAAGTCCTCCCTGCCTGTGCCCT-(BHQ1)	CCAAGCAGCTCCAGCACAG
RSAD2	XM_426208	CTTAAGGAGGCGGGAATGGAG	(FAM)-TTGCTCACAATGCTGACGCTTGGC-(BHQ1)	TTGAACCACCGTTCCCTGATC

## Results

### Clinical observations, serology, and IBV detection in kidney tissues

**Figure 1:**
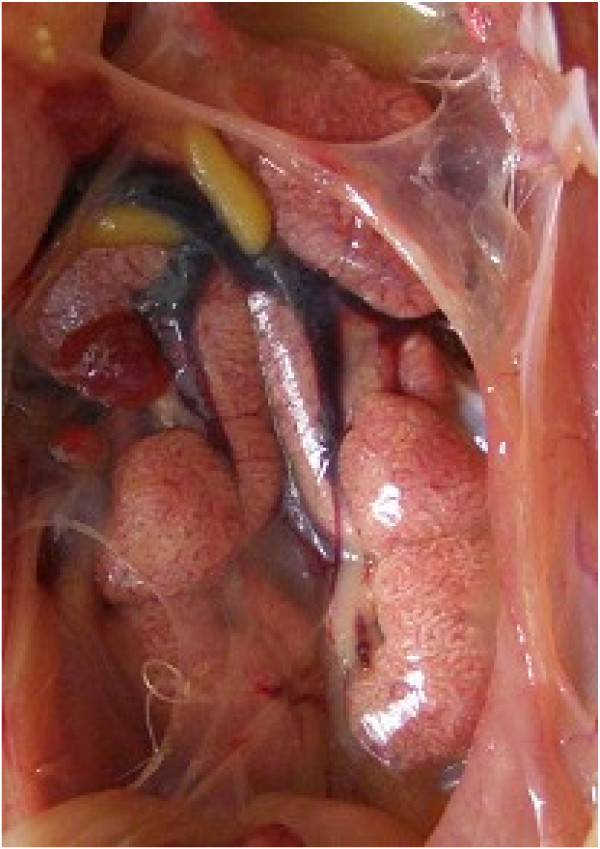
**Renal lesions associated with IB caused by IBV strain ck/CH/LDL/091022.** Note swollen kidneys with tubules and ureters distended with urates.

The chicks inoculated with strain ck/CH/LDL/091022 showed clinical signs from dpi 3 to 13. The diseased chicks were listless, huddled together, and displayed ruffled feathers and dark, shrunken combs. Two chicks died during the experiment and experienced obvious swelling and loss of blood to the kidneys, as well as distended tubules and ureters with urates, suggesting that ck/CH/LDL/091022 was nephropathogenic (Figure 1) [14]. The clinical signs of the inoculated birds tended to gradually disappear after 13 dpi. No obvious clinical signs were observed in the uninfected control chickens during the experiment.

All chickens inoculated with strain ck/CH/LDL/091022 showed a positive serum antibody response at 12 dpi, whereas those in the control group showed a negative serum antibody response. IBV infection was also verified using real-time RT-PCR. Kidney tissue samples from each dead bird had detectable viral RNA with 3.26 × 10^8^ and 1.32 × 10^8^ copies/mg viral RNA, respectively. Viruses were not detected in the kidney samples from the control group. In addition, IBV was recovered from the kidneys of 5 out of 8 chickens challenged with strain ck/CH/LDL/091022 at 15 dpi using 9-day-old embryos; however, virus were not recovered in the kidneys of the eight control birds. Collectively, these results confirmed successful IBV infection of SPF chicken and those used for transcriptome analysis that died from IBV infection.

### Overview of differentially regulated genes after IBV infection

**Figure 2:**
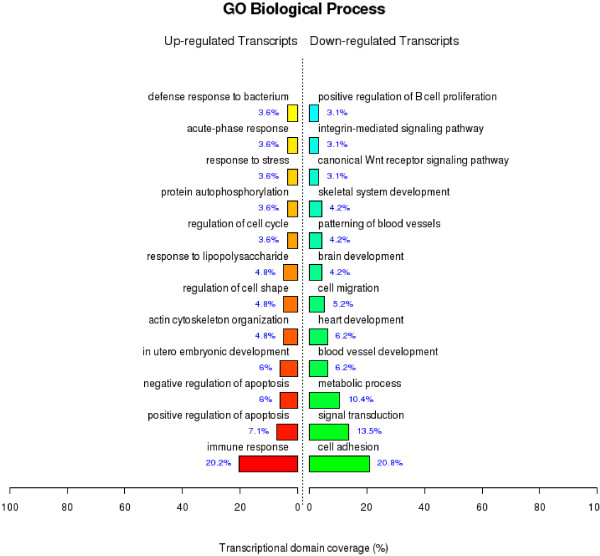
**GO category based on biological processes for differentially expressed genes.** The significant GO category for differentially expressed genes, the DE genes mainly clustered into 12 functional groups with varied numbers. *p*-value < 0.05 and FDR < 0.05 were used as a threshold to select significant GO categories. Exact *p*-value calculation for GO category in Additional file 1.

RNA from kidney tissues retrieved from the two chickens that died at 5 and 6 dpi, respectively, and two uninfected control chickens were extracted and analyzed individually using a microarray to compare gene expression profiles between the groups. Microarray analysis identified 1777 genes, which were differentially regulated more than three-fold in response to IBV infection. Among the differentially expressed (DE) genes, 876 were up-regulated and 901 down-regulated. The DE genes were classified into 98 functional groups (see Additional file 1) according to the GO project for biological processes and the top 12 biological process groups of the genes are shown in Figure 2. The main GO categories for the upregulated genes were immune response (e.g., interleukin (IL)6 and IFN regulatory factor (IRF) 8), positive apoptosis regulation (e.g., BCL2-antagonist/killer 1 and Fas), and negative apoptosis regulation (e.g., clusterin and microphthalmia-associated transcription factor), among others (Figure 2). The primary GO categories for the downregulated genes were response to cell adhesion (e.g., neuropilin 1 and contactin 1), signal transduction (e.g., endoglin and fibrinogen-like 2 ), and metabolic process (e.g., glutathione transferase and arylacetamidedeacetylase-like 4), among others (Figure 2). Particularly, the microarray results indicated that 103 genes associated with immune and inflammatory responses may play important roles in the host defense response during IBV infection (Table 2).

**Table 2:** DE genes associated with immune and inflammatory responses

**Biological process**	**Gene symbol**	**Accession no**	**Gene description**	**Fold change**
**Innate immune response**	MALT1	XM_413722	mucosa associated lymphoid tissue lymphoma translocation gene 1	+2.847583
	CLU	NM_204900	clusterin	+9.196763
	DMBT1	CR353989	deleted in malignant brain tumors 1	+2.036204
	DUSP4	NM_204838	dual specificity phosphatase 4	+19.50851
	DUSP6	NM_204354	dual specificity phosphatase 6	+2.1367393
	DUSP10	NM_001031044	dual specificity phosphatase 10	+2.5124102
	LY96	BX931181	lymphocyte antigen 96	+4.2568717
	SAMHD1	NM_001030845	SAM domain and HD domain 1	+5.7296877
	ERAP1	AJ851612	endoplasmic reticulum aminopeptidase 1	+5.731377
	HMGB1	NM_204902	high mobility group box 1	+3.24355
	TMEM173	CR354327	transmembrane protein 173	+9.81937
	CFI	XM_426329	complement factor I	+5.3699164
	IL1R1	NM_205485	interleukin 1 receptor, type I	+2.814695
	IRAK2	NM_001030605	interleukin-1 receptor-associated kinase 2	+2.1085246
	IRF7	NM_205372	interferon regulatory factor 7	+4.334887
	JUN	NM_001031289	jun proto-oncogene	+3.481312
	MAP3K14	NM_001030927	mitogen-activated protein kinase kinasekinase 14	+2.0599
	MYD88	NM_001030962	myeloid differentiation primary response gene (88)	+2.0273855
	NCF2	CR391316	neutrophil cytosolic factor 2	+9.767744
	NFKB2	NM_204413	nuclear factor of kappa light polypeptide gene enhancer in B-cells 2 (p49/p100)	+4.372285
	MASP1	NM_213586	mannan-binding lectin serine peptidase 1 (C4/C2 activating component of Ra-reactive factor)	-5.984035
	PELI1	NM_001012872	pellino homolog 1 (Drosophila)	-2.6004448
	SIGIRR	NM_001199542	single immunoglobulin and toll-interleukin 1 receptor (TIR) domain	-3.878401
	MRPS6	NM_001031486	mitochondrial ribosomal protein S6	-7.8250012
	IFIH1	NM_001193638	interferon induced with helicase C domain 1	+11.25022
	SRPK1	XM_419265	SFRS protein kinase 1	+2.4798062
	TLR4	NM_001030693	toll-like receptor 4	+2.019901
	TLR5	CR353090	toll-like receptor 5	-2.4051092
	C1R	XM_416518	complement component 1, r subcomponent	+13.635473
	C1S	NM_001030777	complement component 1, s subcomponent	+16.500757
	C3	NM_205405	complement component 3	+25.31715
	C4BPA	NM_204664	complement component 4 binding protein, alpha	-2.476029
	C8B	BX934795	complement component 8, beta polypeptide	-2.8326783
	TRIM25	XM_415653	tripartite motif-containing 25	+3.5009384
	COLEC12	NM_001039599	collectin sub-family member 12	-11.011364
	ITCH	XM_417330	itchy homolog E3 ubiquitin protein ligase (mouse)	+2.3843937
	MARCO	NM_204736	macrophage receptor with collagenous structure	-3.0318317
	FADD	XM_421073	Fas (TNFRSF6)-associated via death domain	-2.127713
	IL18R1	NM_001145225	interleukin 18 receptor 1	+3.5603435
	VNN1	NM_001039288	vanin 1	+30.900112
	IL1RL1	NM_204275	interleukin 1 receptor-like 1	-9.989544
**Cytokine-mediated signaling pathway**	ADAR	AM179858	adenosine deaminase, RNA-specific	+2.2560015
	USP18	CR354286	ubiquitin specific peptidase 18	+14.18105
	EGR1	CR389000	early growth response 1	+4.516151
	IRF8	NM_205416	interferon regulatory factor 8	+4.638908
	IFI35	BX934680	interferon-induced protein 35	+4.3734093
	IFNGR1	NM_001130387	interferon gamma receptor 1	+2.4954834
	IL6	NM_204628	interleukin 6 (interferon, beta 2)	+75.02113
	IL13RA2	NM_001048078	interleukin 13 receptor, alpha 2	+5.0171814
	IRF1	NM_205415	interferon regulatory factor 1	+7.012157
	IRF7	NM_205372	interferon regulatory factor 7	+4.334887
	LIFR	NM_204575	leukemia inhibitory factor receptor alpha	+2.3569908
	MX1	NM_204609	myxovirus (influenza virus) resistance 1, interferon-inducible protein p78 (mouse)	+48.569965
	NCAM1	NM_001242604	neural cell adhesion molecule 1	-7.5168643
	IP6K2	NM_001030596	inositol hexakisphosphate kinase 2	+2.4397569
	PML	XM_413690	promyelocytic leukemia	+3.3186321
	ZC3H15	NM_001006510	zinc finger CCCH-type containing 15	-2.916313
	B2M	NM_001001750	beta-2-microglobulin	+6.515671
	PTPN1	L20630	protein tyrosine phosphatase, non-receptor type 1	+3.4228158
	PTPN2	NM_001199387	protein tyrosine phosphatase, non-receptor type 2	+2.9723573
	CX3CL1	NM_001077232	chemokine (C-X3-C motif) ligand 1	+10.652483
	STAT1	NM_001012914	signal transducer and activator of transcription 1, 91 kDa	+12.110944
	STAT3	NM_001030931	signal transducer and activator of transcription 3 (acute-phase response factor)	+3.900449
	VCAM1	BX950651	vascular cell adhesion molecule 1	-2.8632648
	OASL	NM_205041	2′-5′-oligoadenylate synthetase-like	+101.55924
	SOCS1	NM_001137648	suppressor of cytokine signaling 1	+15.976115
	SOCS3	NM_204600	suppressor of cytokine signaling 3	+30.325502
	CD44	NM_204860	CD44 molecule (Indian blood group)	+5.446098
	CD74	NM_001001613	CD74 molecule, major histocompatibility complex, class II invariant chain	-7.0005507
**Inflammatory response**	CDO1	CR353781	cysteine dioxygenase, type I	-6.4660363
	CEBPB	NM_205253	CCAAT/enhancer binding protein (C/EBP), beta	+3.5429432
	IL23R	XM_422533	interleukin 23 receptor	+3.9536817
	EPHX2	NM_001033645	epoxide hydrolase 2, cytoplasmic	-2.0498822
	LY96	BX931181	lymphocyte antigen 96	+4.2568717
	BLNK	NM_204908	B-cell linker	+3.781344
	AOAH	XM_418835	acyloxyacyl hydrolase (neutrophil)	+3.389245
	AOX1	NM_001038692	aldehyde oxidase 1	-3.0358417
	IGFBP4	NM_204353	insulin-like growth factor binding protein 4	-2.5591307
	IL6	NM_204628	interleukin 6 (interferon, beta 2)	+75.02113
	IRAK2	NM_001030605	interleukin-1 receptor-associated kinase 2	+2.1085246
	KNG1	XM_422766	kininogen 1	-2.1926415
	LIPA	AJ719682	lipase A, lysosomal acid, cholesterol esterase (Wolman disease)	+3.7113423
	LY75	NM_001037836	lymphocyte antigen 75	+3.9913418
	NGFB	M26810	nerve growth factor, beta polypeptide	-2.035398
	NOX4	NM_001101829	NADPH oxidase 4	-9.4226265
	F11R	NM_001083366	F11 receptor	+4.464096
	GAL	NM_001159678	galaninprepropeptide	+8.09671
	SERPINA1	BX932103	serpin peptidase inhibitor, clade A (alpha-1 antiproteinase, antitrypsin), member 1	-30.80776
	S100A9	X61200	S100 calcium binding protein A9	+32.001503
	CCL4	NM_001030360	chemokine (C-C motif) ligand 4	+10.783558
	CCL17	XM_414018	chemokine (C-C motif) ligand 17	+4.898831
	CCL19	BX929857	chemokine (C-C motif) ligand 19	+2.0884924
	CCL20	NM_204438	chemokine (C-C motif) ligand 20	+4.089286
	BMP2	NM_204358	bone morphogenetic protein 2	+3.6641774
	TLR5	CR353090	toll-like receptor 5	-2.4051092
	TNFAIP6	NM_001037837	tumor necrosis factor, alpha-induced protein 6	+51.35508
	C3	NM_205405	complement component 3	+25.31715
	SCG2	BX932277	secretogranin II (chromogranin C)	+3.5667899
	ITCH	XM_417330	itchy homolog E3 ubiquitin protein ligase (mouse)	+2.3843937
	VNN1	NM_001039288	vanin 1	+30.900112
	NMI	BX950337	N-myc (and STAT) interactor	+3.1200686
	CHST2	XR_027120	carbohydrate (N-acetylglucosamine-6-O) sulfotransferase 2	-2.5672126
	HDAC9	CR354257	histone deacetylase 9	-6.544272

Genes were considered significantly up-regulated or down-regulated if the change in their relative expression levels was ≥ 2-fold or ≤ -2-fold.

The DE genes associated with innate immune response, cytokine-mediated signaling pathway, and inflammatory responses were assigned based on GO term. Most genes could also be classified into other categories, but only innate immune response, cytokine-mediated signaling pathway, and inflammatory responses were considered.

+: up-regulated;-: down-regulated.

### Validation of microarray data by real-time RT-PCR

Microarray analysis yields a large amount of data; therefore, it is important to validate differential expression by independent methods. Twelve immune and inflammatory response-related genes with significantly altered expression levels during IBV infection were selected for validation by real-time RT-PCR. The data demonstrated that the overall real-time RT-PCR results were consistent with those of the microarray analysis, although several-fold differences were observed between the two analytical methods because of intrinsic differences between the techniques. The real-time RT-PCR results revealed the same relative regulation pattern of transcription as those of the microarray data, thereby validating and confirming the microarray results, which indicated that the expression levels of many genes were significantly changed in response to IBV infection (Table 3).

**Table 3:** Validation of microarray data by qRT-PCR

**Genes**	**Accession no**	**Microarray fold change (infected/control)**	**Real-time RT-PCR fold change (infected/control)**
ISG12-2	NM_001001296	+141.21	+197.36
SPP1	NM_204535	+19.54	+3.66
IRG1	NM_001030821	+21.26	+4.28
STAT1	NM_001012914	+16.57	+14.60
TNFAIP6	NM_001037837	+136.96	+3.33
IFITM3	XM_420925	+422.64	+234.00
SOCS3	NM_204600	+24.75	+9.86
IL6	NM_204628	+97.97	+490.15
IFIT5	XM_421662	+130.57	+84.17
RSAD2	XM_426208	+165.91	+193.13
MX1	NM_204609	+91.194	+16.60
OASL	NM_205041	+185.05	+22.07

qRT-PCR results of 12 immune and inflammatory response related genes in kidney tissues from IBV-infected chickens compared to those of control chickens. qRT-PCR levels of RNA for a given gene were normalized against the 18S RNA. “+” indicates up-regulated genes.

### Pathway analysis

**Figure 3:**
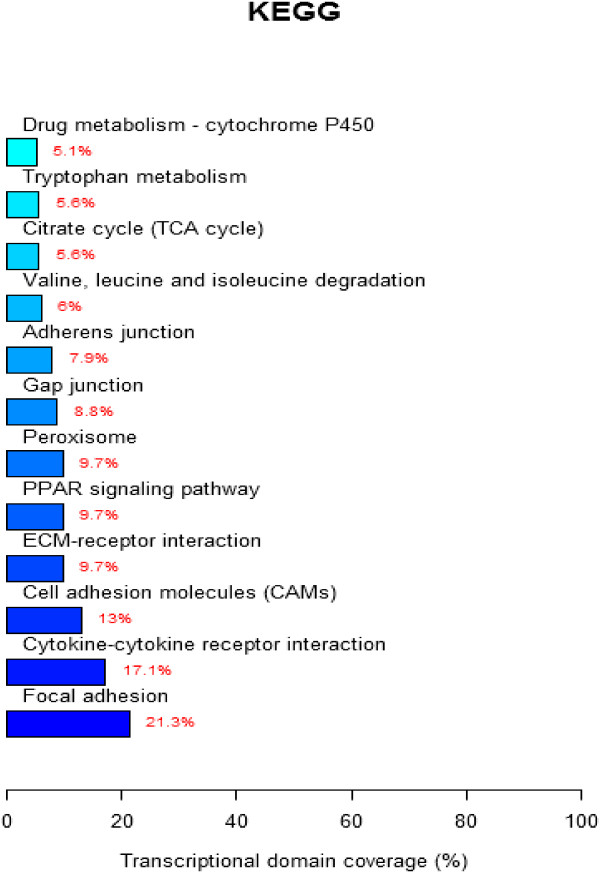
**KEGG pathway analysis for differentially expressed genes.** The significant pathway for differentially expressed genes. A *p*-value < 0.05 and FDR < 0.05 were used as a threshold to select significant KEGG pathways. Exact *p*-value calculation for KEGG pathway analysis in Additional file 2.

To further define DE gene function in chicken kidneys after IBV infection, the KEGG database was used to analyze pathways. The results showed that the DE genes were involved in the focal adhesion pathway, cytokine-cytokine receptor interaction pathway, production of cell adhesion molecules, and peroxisome function as well as other pathways involved in host defense responses against IBV infection (Figure 3).

### STRING analysis of the relationships between DE genes

**Figure 4:**
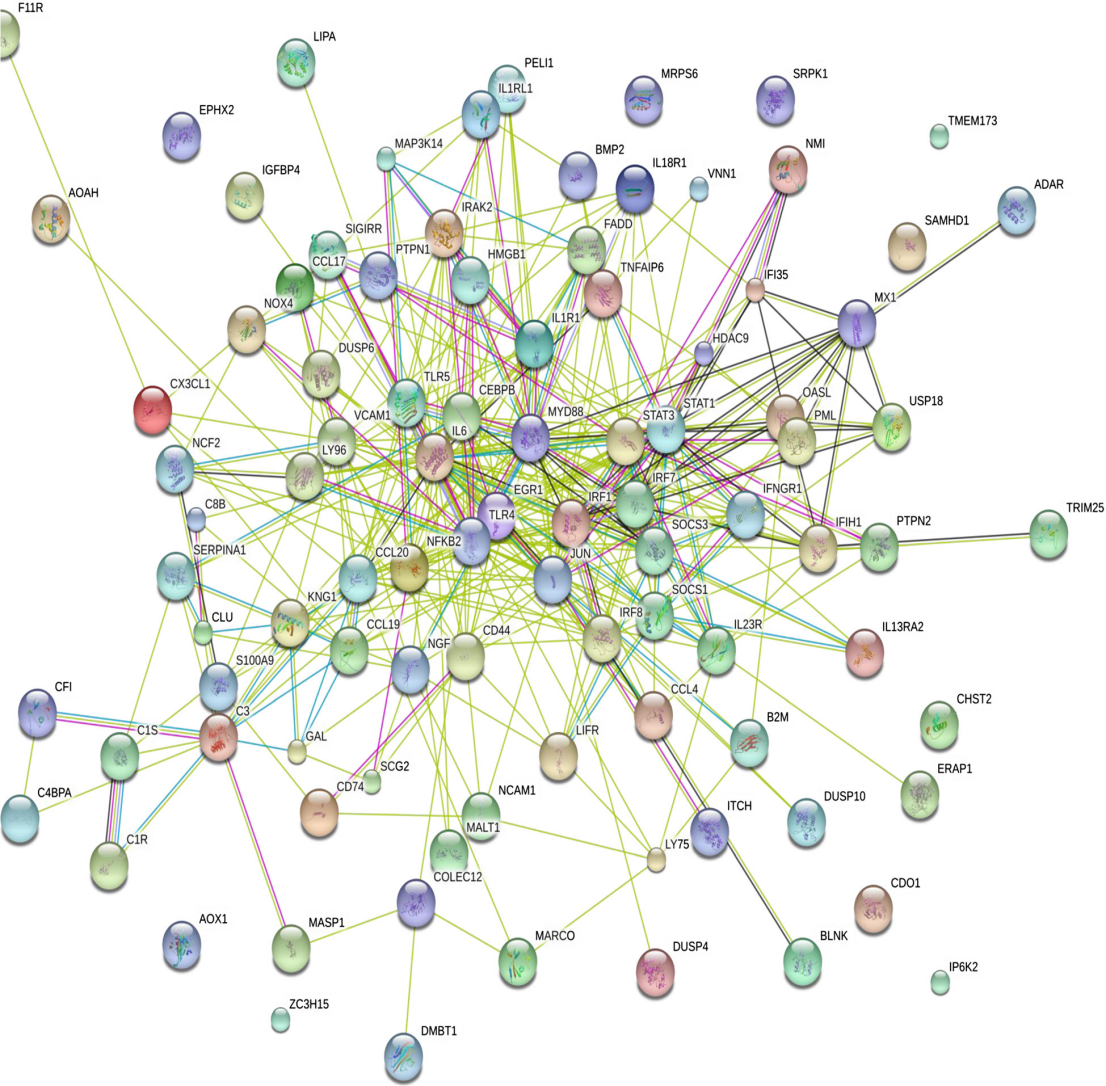
**STRING analysis of the relationship between DE genes related to innate immune response, cytokine-mediated signaling pathways, and inflammatory response related genes.** The network nodes represent the proteins encoded by the DE genes. Seven different colored lines link a number of nodes and represent seven types of evidence used in predicting associations. A red line indicates the presence of fusion evidence; a green line represents neighborhood evidence; a blue line represents co-ocurrence evidence; a purple line represents experimental evidence; a yellow line represents text-mining evidence; a light blue line represents database evidence, and a black line represents co-expression evidence.

The STRING database of known and predicted protein interactions was used to predict interactions of the DE genes listed in Table 2 (innate immune response, cytokine-mediated signaling pathway, and inflammatory response-related genes). Figure 4 summarizes the network of predicted associations for DE gene-encoded proteins. The results indicated that genes IL1RL1, IL1R1, IL-1 receptor-associated kinase 2, TLR4, signal transducer and activator of transcription 1 (STAT1), and myeloid differentiation primary response 88 (MYD88) were associated with many signaling pathways and other immune responses, whereas the genes IRF7, IFN-induced protein with tetratricopeptide repeats 1 (IFIT1), myxovirus resistance 1 (MX1), 2′-5′-oligoadenylate synthetase-like (OASL), IFN gamma receptor 1 (IFNGR1), and suppressor of cytokine signaling (SOCS)3 were also related. IL6, STAT1, MYD88, IRF1, and nuclear factor of kappa light polypeptide gene enhancer in B-cells 2 (NFKB2) were key in the interaction net and linked to SOCS1, SOCS3, STAT3, IRF1, chemokine (C-C motif) ligand (CCL)17, etc., which were further linked to many downstream genes, indicating that all of these genes were inter-related and formed a large network. However, not all genes were linked, indicating that their functions were either unrelated or have not yet been elucidated.

## Discussion

Virulence may differ among IBV strains and, meanwhile, chickens showed various susceptibilities to an IBV strain [17]. To avoid discrepancies, a highly virulent nephropathogenic IBV strain, ck/CH/LDL/091022 [14], was used in this study to infect SPF chickens. Furthermore, fresh kidney tissue samples were collected and used for transcriptome analysis from a chicken infected with ck/CH/LDL/091022 at the time of death. In addition, fresh kidney tissue samples were also collected from of a second chicken infected with ck/CH/LDL/091022 at the time of death, processed individually, and used to confirm the results determined from the first chicken. Only genes that were differentially regulated more than three-fold in both IBV-infected chickens in response to IBV infection were compared to those of control birds and used for further analysis and validation by real-time PCR in this study.

The kidney is a primary target organ of nephropathogenic IBV, in which the transcriptional regulation of host genes after IBV infection can be used as a tool to obtain elaborate insight into virus-host interactions to unravel the pathogenic and/or immune mechanisms of IBV. To our knowledge, this is the first report to use microarray technology to acquire a global profile of host gene expression in chicken kidney cells after IBV infection. Herein, we identified 1777 genes that were differentially regulated more than three-fold compared to the corresponding control group and mostly associated with immune and inflammatory responses. Furthermore, 12 of the immune and inflammatory response DE genes were validated by qRT-PCR. Genes that were differentially expressed during infection can potentially provide insight into the complex regulatory phenomena in response to IBV infection. In our previous study, we identified expression alterations of 53 proteins in kidney tissues of chickens infected with another nephropathogenic IBV strain, ck/CH/LDL/97I, using two-dimensional gel electrophoresis [8,9]. The shared proteins/genes identified by the two methods were mainly involved in oxidoreductase, receptor binding, and transferase activities, whereas some were associated with immune responses. This might be at least due to the different sampling timepoints and the different virulence of viruses used for infecting chickens. The IBV strain ck/CH/LDL/091022 used in this study is highly nephropathogenic and more virulent than ck/CH/LDL/97I [14]. For Newcastle disease virus, it was reported that the more virulent strain persisted longer in the birds and, therefore, was able to increase the magnitude and duration of cell-mediated immunity [18].

Innate immunity provides a first line of defense against pathogens and can be rapidly activated following infection. Activation of the innate immune system relies on the recognition of pathogen-associated molecular patterns (PAMPs) by specific pattern-recognition receptors (PRRs) [19]. TLR4, TLR5, TLR15, and TLR16 belong to the TLR family and are involved in sensing and initiating immune responses to viral infection [20]. All of the TLRs were significantly up-regulated after IBV infection. Reportedly, severe acute respiratory syndrome (SARS-CoV) and mouse hepatitis virus (MHV) infection can also induce TLR4 expression [21], suggesting that TLR4 possibly has a similar effect both in avian and mammalian species as well as during coronavirus infection. Similar to the present results, a previous study reported that the transcription level of TLR15, a poultry-specific TLR, was significantly up-regulated after infection with Marek’s disease virus [22], infectious bursa disease virus infection [23], and avian influenza virus H9N2-infected chickens in the lungs [24], implicating a similar role of TLR15 in sensing and initiating responses to viruses after infection.

Melanoma differentiation-associated gene-5 (MDA5 or IFIH1) belongs to the retinoic acid-inducible-like helicase family of PRRs and sense viral RNA in the cytoplasm. In this study, MDA5 expression levels were significantly increased after IBV infection. SARS-Cov infection can also induce MDA5 expression in vitro and MHV is recognized by MDA5 in brain macrophages, oligodendrocytes, and microglial cells [25-27], suggesting a role of chicken MDA5 against IBV infection and a possible correlation with induction of the inflammatory response.

Although distinct microbial PAMPs activate different TLRs, they ultimately cross paths during the transcriptional activation of IRF3, IRF7, and NF-kB, all of which translocate to the nucleus and activate transcription of type I IFN (IFN-α and IFN-β) [28] and the subsequent IFN-enhanced production of IFN-stimulated genes (ISGs). IBV can induce acute IFN-α production [29] and the ability to induce IFN is linked to the virulence and adaptability of the IBV strain to a particular host system [30,31]. In this study, IFN expression was not significantly upregulated after IBV infection; however, some IRFs and ISGs, such as IRF1, IRF7, IRF8, IRF10, NF-kB, STAT1, MyD88, OSAL, MX1, IFIT5, ISG12-2, RSAD2, IFI35, and IFI27L2 were all up-regulated. Therefore, we hypothesized that IFN might be induced during early infection, as indicated by the subsequent enhanced production of ISGs because increased expression of a variety of ISGs, such as OSAL, MX1, IFIT5, ISG12-2, RSAD2, IFI35, protein kinase R (PKR), and IFI27L2, was found in this study. STAT proteins form homodimeric and heterodimeric complexes to activate transcription of some ISGs [32]. Similar to the results in the current study, a previous report showed that STAT1 expression was significantly increased response to IBV infection in *ovo*[10], suggesting that IBV infection can activate the JAK-STAT pathway and activate transcription of ISGs. MX1 is a dynamin-like large guanosinetriphosphatase (GTPase), which has antiviral activity against a wide range of RNA viruses [33]. PKR (also known as EIF2αK2) is constitutively expressed as an inactive kinase that is activated by viral double-stranded RNA and plays an important role in the cellular antiviral response pathway. OASL is a member of the OAS gene family, which in combination with RNaseL constitutes an antiviral RNA decay pathway [34]. The activation of both OAS and PKR results in global degradation of cellular RNA and translation inhibition, which may also inhibit viral propagation [28]. In addition, we found that the MxGTPase pathway, the protein kinase R pathway, and the 2′-5′ oligoadenylate-synthetase-directed ribonuclease L pathway might be involved in anti-IBV infection, but the ISG15 ubiquitin-like pathway was not. These pathways might exert their effects through different mechanisms of action, such as direct targeting of viral entry, inhibition of protein synthesis, or degradation of viral RNA [35], and play an important role in anti-IBV responses and host defense during IBV infection.

Viral infections induce a pro-inflammatory response that include cytokine and chemokine expression [36]. In this study, increased expression of IL6, IL18, IL10RA, IL17RA, CCL4, CCL20, CCL17, and CCL19 was observed after IBV infection; however, chemokine (C-X-C motif) ligand 12 expression was found to be decreased. IL6 was also up-regulated during other nidovirus infections, such as SARS-CoV, porcine respiratory coronavirus, equine arteritis virus, and transmissible gastroenteritis coronavirus (TGEV) [37-39]. These findings suggested that IL6, IL18, and IL10 may have similar functions in IBV infection as those in other coronavirus infections. The important negative regulators SOCS-1 and SOCS-3 were significantly up-regulated in this study. SOCS-1 and SOCS-3 participate in the feedback system of IL6 signal transduction by binding to phosphorylated tyrosine residues of a component of the IL6 receptor gp130 [40]. The observed changes in expression of IL6, SOCS-1, and SOCS-3 in kidney tissues of IBV-infected chickens suggested a balance between pro-and anti-inflammatory cytokines, which seemed to be critical for IBV pathogenesis.

Our data also presented an interesting set of apoptosis-related genes after IBV infection. In this study, the expression of the Fas, caspase (casp) 1, casp9, casp18, Bcl-2L1, BCL2-antagonist/killer 1, myeloid cell leukemia sequence 1 (Mcl-1), and eukaryotic initiation factor 2a genes was significantly increased compared to the control group. The caspase proteins are executioners of apoptosis [41]. Extrinsic signals to initiate the apoptotic pathways from receptors (e.g., Fas ligand) culminate in the activation of caspase 8, which activates the effector caspase 3, while intrinsic signaling requires the participation of the mitochondria in releasing cytochrome c to activate caspase 9 for the downstream activation of caspase 3. Both pro-apoptotic (e.g., Bax and Bak) and anti-apoptotic proteins (e.g., Bcl-2 and Bcl-XL) from the Bcl-2 family are key proteins in the intrinsic apoptosis signaling pathway [28]. Recent reports have suggested that IBV also triggers apoptosis during the late stages of its cytolytic infection cycle. Similar results were found in IBV-infected Vero cells at 24 h postinfection, revealing an up-regulation at the transcriptional level of both pro-apoptotic Bak and pro-survival Mcl-1, which may play essential roles in maintaining the intricate balance between life and death of infected cells to ensure a successful infection cycle [42]. Taken together, it can be hypothesized that IBV activates apoptosis through Fas/FasL activation and mitochondrial-dependent pathways, similar to that of TGEV [43,44]. Furthermore, Bak and Mcl-1 may play key roles in the regulation of IBV-induced apoptosis, not only at an early stage of infection in vitro, but also at a late stage of infection in vivo. In other words, apoptosis is a nonspecific defense mechanism against IBV infection through abortion of viral multiplication by premature lysis of infected cells.

In addition, several other immune function-related genes were up-regulated, including major histocompatibility complex (MHC) class I antigen, MHC BF2 class I, MR1, MHC BF1 class I, immunoresponsive 1 homolog, secreted phosphoprotein 1, the complement system (C3, C1S, C4 and C1R), among other genes involved in the IFR response (IRF1,IRF7,IRF8, IFIH1, IFIT-like ISG12-2, and IFITM3 ). The above results indicated that both adaptive and innate immunity responded to IBV infection and the complement system might be a functional bridge between innate and adaptive immune responses to allow an integrated host defense to pathogenic challenges [45].

The data in this study were obtained from kidney of chickens infected with a nephropathogenic IBV strain at 5 and 6 dpi, respectively, concomitant with the peak of the virus titers in the kidney [14]. The analysis of the global profile of host gene expression provided a good overview of host response to IBV infection and is helpful for understanding IBV mechanism of disease or death. The results also provided basic information needed to extend our understanding of the nature of the virus-host interactions. However, analysis of the innate host immune response in kidneys at those time points may not be sufficient to completely understand IBV immunologic mechanisms. In addition, the roles of individual cell types in this response cannot be effectively measured in this way. Therefore, further analysis of the above-mentioned concerns is warranted. Given the consistent utilization of current vaccines and the continued emergence of new virulent IBV genotypes throughout the world, the development of better vaccines and control strategies will require a greater understanding of pathogenic mechanisms.

## Conclusions

We screened and identified differentially expressed transcripts in IBV-infected chicken kidney tissues using microarray analysis. A total of 103 DE genes were identified in this study, which were associated with immune and inflammatory responses, as well as the cytokine-mediated signaling pathway; therefore, these genes may play major roles in the host defense response or/and IBV pathogenesis. Combining network analysis with differential gene expression analysis helped to uncover high-confidence genes, such as IL6, TLR4, STAT1, MYD88, IRF1, and NFKB2, and several immune-related genes, such as IRF7, IFIT1, MX1, OASL, IFNGR1, and SOCS3, that have potentially important consequences to IBV infection. Our results should prove helpful to elucidate the pathogenic mechanisms of IBV and the host antiviral response.

## Abbreviations

IBV: Infectious bronchitis virus; IB: Infectious bronchitis; TLR: Toll-like receptors; Mx: IFN-induced antiviral genes; SPF: Specific pathogen free; Dpi: Day post infection; qRT-PCR: Quantitative real-time RT-PCR; DE: Differential expression; PRRs: Pattern recognition receptors; PAMPs: Pathogen associated molecular patterns; MHV: Mouse hepatitis virus; MDA5: Melanoma differentiation-associated gene-5; ISGs: IFN-stimulated genes; TGEV: Transmissible gastroenteritis coronavirus.

## Competing interests

The authors declare that they have no competing interests.

## Authors’ contributions

SL designed the study. SL and FC drafted the manuscript. FC, XL, ZH, and YS performed virus infection and tests for the presence of IBV. FC made substantial contributions to the bioinformatics and statistical analysis. FC and XL confirmed differential gene expression by real-time RT-PCR. SL wrote the manuscript. SL and XK revised the manuscript. All authors read and approved the final manuscript.

## Supplementary Material

Additional file 1 The complete microarray dataset of the DE genes with GO biological process annotations. Click here for file

Additional file 2 **The *p*-value of KEGG pathway analysis.** Click here for file
