# Supplementary material for: Transcriptome analysis of chicken kidney tissues following coronavirus avian infectious bronchitis virus infection
Source: BMC Genomics. 2013 Oct 30;14:743. doi: 10.1186/1471-2164-14-743 (PMC3870970; doi:10.1186/1471-2164-14-743)
Supplement: Additional file 1 — The complete microarray dataset of the DE genes with GO biological process annotations. [file 1471-2164-14-743-S1.doc]

**Additional file 1**

The complete microarray dataset of DE genes with GO biological process annotations

| **Rank** | **ID** | **Term** | **List**  **Hits** | **List**  **Total** | **Population**  **Hits** | **Population**  **Total** | **P-value** |
| --- | --- | --- | --- | --- | --- | --- | --- |
| Upregulated transcripts | | | | | | | |
| 1 | [GO:0006955](http://www.ebi.ac.uk/ego/DisplayGoTerm?id=GO:0006955) | [immune response](http://www.ebi.ac.uk/ego/DisplayGoTerm?id=GO:0006955) | 17 | 171 | 88 | 5693 | 5.73e-10 |
| 2 | [GO:0043065](http://www.ebi.ac.uk/ego/DisplayGoTerm?id=GO:0043065) | [positive regulation of apoptosis](http://www.ebi.ac.uk/ego/DisplayGoTerm?id=GO:0043065) | 6 | 171 | 45 | 5693 | 2.07e-03 |
| 3 | [GO:0043066](http://www.ebi.ac.uk/ego/DisplayGoTerm?id=GO:0043066) | [negative regulation of apoptosis](http://www.ebi.ac.uk/ego/DisplayGoTerm?id=GO:0043066) | 5 | 171 | 63 | 5693 | 4.00e-02 |
| 4 | [GO:0001701](http://www.ebi.ac.uk/ego/DisplayGoTerm?id=GO:0001701) | [in utero embryonic development](http://www.ebi.ac.uk/ego/DisplayGoTerm?id=GO:0001701) | 5 | 171 | 62 | 5693 | 3.77e-02 |
| 5 | [GO:0030036](http://www.ebi.ac.uk/ego/DisplayGoTerm?id=GO:0030036) | [actin cytoskeleton organization](http://www.ebi.ac.uk/ego/DisplayGoTerm?id=GO:0030036) | 4 | 171 | 32 | 5693 | 1.47e-02 |
| 6 | [GO:0008360](http://www.ebi.ac.uk/ego/DisplayGoTerm?id=GO:0008360) | [regulation of cell shape](http://www.ebi.ac.uk/ego/DisplayGoTerm?id=GO:0008360) | 4 | 171 | 14 | 5693 | 6.21e-04 |
| 7 | [GO:0032496](http://www.ebi.ac.uk/ego/DisplayGoTerm?id=GO:0032496) | [response to lipopolysaccharide](http://www.ebi.ac.uk/ego/DisplayGoTerm?id=GO:0032496) | 4 | 171 | 22 | 5693 | 3.76e-03 |
| 8 | [GO:0051726](http://www.ebi.ac.uk/ego/DisplayGoTerm?id=GO:0051726) | [regulation of cell cycle](http://www.ebi.ac.uk/ego/DisplayGoTerm?id=GO:0051726) | 3 | 171 | 19 | 5693 | 1.81e-02 |
| 9 | [GO:0046777](http://www.ebi.ac.uk/ego/DisplayGoTerm?id=GO:0046777) | [protein autophosphorylation](http://www.ebi.ac.uk/ego/DisplayGoTerm?id=GO:0046777) | 3 | 171 | 17 | 5693 | 1.33e-02 |
| 10 | [GO:0006950](http://www.ebi.ac.uk/ego/DisplayGoTerm?id=GO:0006950) | [response to stress](http://www.ebi.ac.uk/ego/DisplayGoTerm?id=GO:0006950) | 3 | 171 | 23 | 5693 | 3.03e-02 |
| 11 | [GO:0006953](http://www.ebi.ac.uk/ego/DisplayGoTerm?id=GO:0006953) | [acute-phase response](http://www.ebi.ac.uk/ego/DisplayGoTerm?id=GO:0006953) | 3 | 171 | 4 | 5693 | 1.04e-04 |
| 12 | [GO:0042742](http://www.ebi.ac.uk/ego/DisplayGoTerm?id=GO:0042742) | [defense response to bacterium](http://www.ebi.ac.uk/ego/DisplayGoTerm?id=GO:0042742) | 3 | 171 | 25 | 5693 | 3.77e-02 |
| 13 | [GO:0050853](http://www.ebi.ac.uk/ego/DisplayGoTerm?id=GO:0050853) | [B cell receptor signaling pathway](http://www.ebi.ac.uk/ego/DisplayGoTerm?id=GO:0050853) | 3 | 171 | 9 | 5693 | 1.96e-03 |
| 14 | [GO:0030217](http://www.ebi.ac.uk/ego/DisplayGoTerm?id=GO:0030217) | [T cell differentiation](http://www.ebi.ac.uk/ego/DisplayGoTerm?id=GO:0030217) | 2 | 171 | 6 | 5693 | 1.24e-02 |
| 15 | [GO:0000082](http://www.ebi.ac.uk/ego/DisplayGoTerm?id=GO:0000082) | [G1/S transition of mitotic cell cycle](http://www.ebi.ac.uk/ego/DisplayGoTerm?id=GO:0000082) | 2 | 171 | 10 | 5693 | 3.44e-02 |
| 16 | [GO:0001708](http://www.ebi.ac.uk/ego/DisplayGoTerm?id=GO:0001708) | [cell fate specification](http://www.ebi.ac.uk/ego/DisplayGoTerm?id=GO:0001708) | 2 | 171 | 12 | 5693 | 4.86e-02 |
| 17 | [GO:0048008](http://www.ebi.ac.uk/ego/DisplayGoTerm?id=GO:0048008) | [platelet-derived growth factor receptor signaling pathway](http://www.ebi.ac.uk/ego/DisplayGoTerm?id=GO:0048008) | 2 | 171 | 8 | 5693 | 2.23e-02 |
| 18 | [GO:0060445](http://www.ebi.ac.uk/ego/DisplayGoTerm?id=GO:0060445) | [branching involved in salivary gland morphogenesis](http://www.ebi.ac.uk/ego/DisplayGoTerm?id=GO:0060445) | 2 | 171 | 6 | 5693 | 1.24e-02 |
| 19 | [GO:0032869](http://www.ebi.ac.uk/ego/DisplayGoTerm?id=GO:0032869) | [cellular response to insulin stimulus](http://www.ebi.ac.uk/ego/DisplayGoTerm?id=GO:0032869) | 2 | 171 | 10 | 5693 | 3.44e-02 |
| 20 | [GO:0010811](http://www.ebi.ac.uk/ego/DisplayGoTerm?id=GO:0010811) | [positive regulation of cell-substrate adhesion](http://www.ebi.ac.uk/ego/DisplayGoTerm?id=GO:0010811) | 2 | 171 | 5 | 5693 | 8.45e-03 |
| 21 | [GO:0009636](http://www.ebi.ac.uk/ego/DisplayGoTerm?id=GO:0009636) | [response to toxin](http://www.ebi.ac.uk/ego/DisplayGoTerm?id=GO:0009636) | 2 | 171 | 7 | 5693 | 1.71e-02 |
| 22 | [GO:0040008](http://www.ebi.ac.uk/ego/DisplayGoTerm?id=GO:0040008) | [regulation of growth](http://www.ebi.ac.uk/ego/DisplayGoTerm?id=GO:0040008) | 2 | 171 | 10 | 5693 | 3.44e-02 |
| 23 | [GO:0009792](http://www.ebi.ac.uk/ego/DisplayGoTerm?id=GO:0009792) | [embryo development ending in birth or egg hatching](http://www.ebi.ac.uk/ego/DisplayGoTerm?id=GO:0009792) | 2 | 171 | 10 | 5693 | 3.44e-02 |
| 24 | [GO:0001706](http://www.ebi.ac.uk/ego/DisplayGoTerm?id=GO:0001706) | [endoderm formation](http://www.ebi.ac.uk/ego/DisplayGoTerm?id=GO:0001706) | 2 | 171 | 3 | 5693 | 2.64e-03 |
| 25 | [GO:0042113](http://www.ebi.ac.uk/ego/DisplayGoTerm?id=GO:0042113) | [B cell activation](http://www.ebi.ac.uk/ego/DisplayGoTerm?id=GO:0042113) | 2 | 171 | 8 | 5693 | 2.23e-02 |
| 26 | [GO:0042074](http://www.ebi.ac.uk/ego/DisplayGoTerm?id=GO:0042074) | [cell migration involved in gastrulation](http://www.ebi.ac.uk/ego/DisplayGoTerm?id=GO:0042074) | 2 | 171 | 5 | 5693 | 8.45e-03 |
| 27 | [GO:0009617](http://www.ebi.ac.uk/ego/DisplayGoTerm?id=GO:0009617) | [response to bacterium](http://www.ebi.ac.uk/ego/DisplayGoTerm?id=GO:0009617) | 2 | 171 | 3 | 5693 | 2.64e-03 |
| 28 | [GO:0031532](http://www.ebi.ac.uk/ego/DisplayGoTerm?id=GO:0031532) | [actin cytoskeleton reorganization](http://www.ebi.ac.uk/ego/DisplayGoTerm?id=GO:0031532) | 2 | 171 | 5 | 5693 | 8.45e-03 |
| 29 | [GO:0050832](http://www.ebi.ac.uk/ego/DisplayGoTerm?id=GO:0050832) | [defense response to fungus](http://www.ebi.ac.uk/ego/DisplayGoTerm?id=GO:0050832) | 2 | 171 | 3 | 5693 | 2.64e-03 |
| 30 | [GO:0051607](http://www.ebi.ac.uk/ego/DisplayGoTerm?id=GO:0051607) | [defense response to virus](http://www.ebi.ac.uk/ego/DisplayGoTerm?id=GO:0051607) | 2 | 171 | 8 | 5693 | 2.23e-02 |
| 31 | [GO:0060324](http://www.ebi.ac.uk/ego/DisplayGoTerm?id=GO:0060324) | [face development](http://www.ebi.ac.uk/ego/DisplayGoTerm?id=GO:0060324) | 2 | 171 | 6 | 5693 | 1.24e-02 |
| 32 | [GO:0003161](http://www.ebi.ac.uk/ego/DisplayGoTerm?id=GO:0003161) | [cardiac conduction system development](http://www.ebi.ac.uk/ego/DisplayGoTerm?id=GO:0003161) | 1 | 171 | 1 | 5693 | 3.00e-02 |
| 33 | [GO:0033233](http://www.ebi.ac.uk/ego/DisplayGoTerm?id=GO:0033233) | [regulation of protein sumoylation](http://www.ebi.ac.uk/ego/DisplayGoTerm?id=GO:0033233) | 1 | 171 | 1 | 5693 | 3.00e-02 |
| 34 | [GO:0002548](http://www.ebi.ac.uk/ego/DisplayGoTerm?id=GO:0002548) | [monocyte chemotaxis](http://www.ebi.ac.uk/ego/DisplayGoTerm?id=GO:0002548) | 1 | 171 | 1 | 5693 | 3.00e-02 |
| 35 | [GO:0019100](http://www.ebi.ac.uk/ego/DisplayGoTerm?id=GO:0019100) | [male germ-line sex determination](http://www.ebi.ac.uk/ego/DisplayGoTerm?id=GO:0019100) | 1 | 171 | 1 | 5693 | 3.00e-02 |
| 36 | [GO:0030502](http://www.ebi.ac.uk/ego/DisplayGoTerm?id=GO:0030502) | [negative regulation of bone mineralization](http://www.ebi.ac.uk/ego/DisplayGoTerm?id=GO:0030502) | 1 | 171 | 1 | 5693 | 3.00e-02 |
| 37 | [GO:0060350](http://www.ebi.ac.uk/ego/DisplayGoTerm?id=GO:0060350) | [endochondral bone morphogenesis](http://www.ebi.ac.uk/ego/DisplayGoTerm?id=GO:0060350) | 1 | 171 | 1 | 5693 | 3.00e-02 |
| 38 | [GO:0060517](http://www.ebi.ac.uk/ego/DisplayGoTerm?id=GO:0060517) | [epithelial cell proliferation involved in prostatic bud elongation](http://www.ebi.ac.uk/ego/DisplayGoTerm?id=GO:0060517) | 1 | 171 | 1 | 5693 | 3.00e-02 |
| 39 | [GO:0043549](http://www.ebi.ac.uk/ego/DisplayGoTerm?id=GO:0043549) | [regulation of kinase activity](http://www.ebi.ac.uk/ego/DisplayGoTerm?id=GO:0043549) | 1 | 171 | 1 | 5693 | 3.00e-02 |
| 40 | [GO:0042489](http://www.ebi.ac.uk/ego/DisplayGoTerm?id=GO:0042489) | [negative regulation of odontogenesis of dentine-containing tooth](http://www.ebi.ac.uk/ego/DisplayGoTerm?id=GO:0042489) | 1 | 171 | 1 | 5693 | 3.00e-02 |
| 41 | [GO:0044419](http://www.ebi.ac.uk/ego/DisplayGoTerm?id=GO:0044419) | [interspecies interaction between organisms](http://www.ebi.ac.uk/ego/DisplayGoTerm?id=GO:0044419) | 1 | 171 | 1 | 5693 | 3.00e-02 |
| 42 | [GO:0046628](http://www.ebi.ac.uk/ego/DisplayGoTerm?id=GO:0046628) | [positive regulation of insulin receptor signaling pathway](http://www.ebi.ac.uk/ego/DisplayGoTerm?id=GO:0046628) | 1 | 171 | 1 | 5693 | 3.00e-02 |
| 43 | [GO:0006922](http://www.ebi.ac.uk/ego/DisplayGoTerm?id=GO:0006922) | [cleavage of lamin](http://www.ebi.ac.uk/ego/DisplayGoTerm?id=GO:0006922) | 1 | 171 | 1 | 5693 | 3.00e-02 |
| 44 | [GO:0032078](http://www.ebi.ac.uk/ego/DisplayGoTerm?id=GO:0032078) | [negative regulation of endodeoxyribonuclease activity](http://www.ebi.ac.uk/ego/DisplayGoTerm?id=GO:0032078) | 1 | 171 | 1 | 5693 | 3.00e-02 |
| 45 | [GO:0051354](http://www.ebi.ac.uk/ego/DisplayGoTerm?id=GO:0051354) | [negative regulation of oxidoreductase activity](http://www.ebi.ac.uk/ego/DisplayGoTerm?id=GO:0051354) | 1 | 171 | 1 | 5693 | 3.00e-02 |
| 46 | [GO:0006926](http://www.ebi.ac.uk/ego/DisplayGoTerm?id=GO:0006926) | [virus-infected cell apoptosis](http://www.ebi.ac.uk/ego/DisplayGoTerm?id=GO:0006926) | 1 | 171 | 1 | 5693 | 3.00e-02 |
| 47 | [GO:0002682](http://www.ebi.ac.uk/ego/DisplayGoTerm?id=GO:0002682) | [regulation of immune system process](http://www.ebi.ac.uk/ego/DisplayGoTerm?id=GO:0002682) | 1 | 171 | 1 | 5693 | 3.00e-02 |
| 48 | [GO:0033572](http://www.ebi.ac.uk/ego/DisplayGoTerm?id=GO:0033572) | [transferrin transport](http://www.ebi.ac.uk/ego/DisplayGoTerm?id=GO:0033572) | 1 | 171 | 1 | 5693 | 3.00e-02 |
| 49 | [GO:0002377](http://www.ebi.ac.uk/ego/DisplayGoTerm?id=GO:0002377) | [immunoglobulin production](http://www.ebi.ac.uk/ego/DisplayGoTerm?id=GO:0002377) | 1 | 171 | 1 | 5693 | 3.00e-02 |
| 50 | [GO:0010940](http://www.ebi.ac.uk/ego/DisplayGoTerm?id=GO:0010940) | [positive regulation of necrotic cell death](http://www.ebi.ac.uk/ego/DisplayGoTerm?id=GO:0010940) | 1 | 171 | 1 | 5693 | 3.00e-02 |
| 51 | [GO:0045619](http://www.ebi.ac.uk/ego/DisplayGoTerm?id=GO:0045619) | [regulation of lymphocyte differentiation](http://www.ebi.ac.uk/ego/DisplayGoTerm?id=GO:0045619) | 1 | 171 | 1 | 5693 | 3.00e-02 |
| 52 | [GO:0045637](http://www.ebi.ac.uk/ego/DisplayGoTerm?id=GO:0045637) | [regulation of myeloid cell differentiation](http://www.ebi.ac.uk/ego/DisplayGoTerm?id=GO:0045637) | 1 | 171 | 1 | 5693 | 3.00e-02 |
| 53 | [GO:0050869](http://www.ebi.ac.uk/ego/DisplayGoTerm?id=GO:0050869) | [negative regulation of B cell activation](http://www.ebi.ac.uk/ego/DisplayGoTerm?id=GO:0050869) | 1 | 171 | 1 | 5693 | 3.00e-02 |
| 54 | [GO:0060008](http://www.ebi.ac.uk/ego/DisplayGoTerm?id=GO:0060008) | [Sertoli cell differentiation](http://www.ebi.ac.uk/ego/DisplayGoTerm?id=GO:0060008) | 1 | 171 | 1 | 5693 | 3.00e-02 |
| 55 | [GO:0060708](http://www.ebi.ac.uk/ego/DisplayGoTerm?id=GO:0060708) | [spongiotrophoblast differentiation](http://www.ebi.ac.uk/ego/DisplayGoTerm?id=GO:0060708) | 1 | 171 | 1 | 5693 | 3.00e-02 |
| 56 | [GO:0072040](http://www.ebi.ac.uk/ego/DisplayGoTerm?id=GO:0072040) | [negative regulation of mesenchymal stem cell apoptosis involved in nephron morphogenesis](http://www.ebi.ac.uk/ego/DisplayGoTerm?id=GO:0072040) | 1 | 171 | 1 | 5693 | 3.00e-02 |
| 57 | [GO:0072133](http://www.ebi.ac.uk/ego/DisplayGoTerm?id=GO:0072133) | [metanephric mesenchyme morphogenesis](http://www.ebi.ac.uk/ego/DisplayGoTerm?id=GO:0072133) | 1 | 171 | 1 | 5693 | 3.00e-02 |
| 58 | [GO:0072134](http://www.ebi.ac.uk/ego/DisplayGoTerm?id=GO:0072134) | [nephrogenic mesenchyme morphogenesis](http://www.ebi.ac.uk/ego/DisplayGoTerm?id=GO:0072134) | 1 | 171 | 1 | 5693 | 3.00e-02 |
| 59 | [GO:0001552](http://www.ebi.ac.uk/ego/DisplayGoTerm?id=GO:0001552) | [ovarian follicle atresia](http://www.ebi.ac.uk/ego/DisplayGoTerm?id=GO:0001552) | 1 | 171 | 1 | 5693 | 3.00e-02 |
| 60 | [GO:0032463](http://www.ebi.ac.uk/ego/DisplayGoTerm?id=GO:0032463) | [negative regulation of protein homooligomerization](http://www.ebi.ac.uk/ego/DisplayGoTerm?id=GO:0032463) | 1 | 171 | 1 | 5693 | 3.00e-02 |
| 61 | [GO:0051788](http://www.ebi.ac.uk/ego/DisplayGoTerm?id=GO:0051788) | [response to misfolded protein](http://www.ebi.ac.uk/ego/DisplayGoTerm?id=GO:0051788) | 1 | 171 | 1 | 5693 | 3.00e-02 |
| 62 | [GO:0045670](http://www.ebi.ac.uk/ego/DisplayGoTerm?id=GO:0045670) | [regulation of osteoclast differentiation](http://www.ebi.ac.uk/ego/DisplayGoTerm?id=GO:0045670) | 1 | 171 | 1 | 5693 | 3.00e-02 |
| 63 | [GO:0046881](http://www.ebi.ac.uk/ego/DisplayGoTerm?id=GO:0046881) | [positive regulation of follicle-stimulating hormone secretion](http://www.ebi.ac.uk/ego/DisplayGoTerm?id=GO:0046881) | 1 | 171 | 1 | 5693 | 3.00e-02 |
| 64 | [GO:0048178](http://www.ebi.ac.uk/ego/DisplayGoTerm?id=GO:0048178) | [negative regulation of hepatocyte growth factor biosynthetic process](http://www.ebi.ac.uk/ego/DisplayGoTerm?id=GO:0048178) | 1 | 171 | 1 | 5693 | 3.00e-02 |
| 65 | [GO:0046654](http://www.ebi.ac.uk/ego/DisplayGoTerm?id=GO:0046654) | [tetrahydrofolate biosynthetic process](http://www.ebi.ac.uk/ego/DisplayGoTerm?id=GO:0046654) | 1 | 171 | 1 | 5693 | 3.00e-02 |
| 66 | [GO:0060334](http://www.ebi.ac.uk/ego/DisplayGoTerm?id=GO:0060334) | [regulation of interferon-gamma-mediated signaling pathway](http://www.ebi.ac.uk/ego/DisplayGoTerm?id=GO:0060334) | 1 | 171 | 1 | 5693 | 3.00e-02 |
| 67 | [GO:0060338](http://www.ebi.ac.uk/ego/DisplayGoTerm?id=GO:0060338) | [regulation of type I interferon-mediated signaling pathway](http://www.ebi.ac.uk/ego/DisplayGoTerm?id=GO:0060338) | 1 | 171 | 1 | 5693 | 3.00e-02 |
| 68 | [GO:0051016](http://www.ebi.ac.uk/ego/DisplayGoTerm?id=GO:0051016) | [barbed-end actin filament capping](http://www.ebi.ac.uk/ego/DisplayGoTerm?id=GO:0051016) | 1 | 171 | 1 | 5693 | 3.00e-02 |
| 69 | [GO:0060982](http://www.ebi.ac.uk/ego/DisplayGoTerm?id=GO:0060982) | [coronary artery morphogenesis](http://www.ebi.ac.uk/ego/DisplayGoTerm?id=GO:0060982) | 1 | 171 | 1 | 5693 | 3.00e-02 |
| 70 | [GO:0045358](http://www.ebi.ac.uk/ego/DisplayGoTerm?id=GO:0045358) | [negative regulation of interferon-beta biosynthetic process](http://www.ebi.ac.uk/ego/DisplayGoTerm?id=GO:0045358) | 1 | 171 | 1 | 5693 | 3.00e-02 |
| 71 | [GO:0050689](http://www.ebi.ac.uk/ego/DisplayGoTerm?id=GO:0050689) | [negative regulation of defense response to virus by host](http://www.ebi.ac.uk/ego/DisplayGoTerm?id=GO:0050689) | 1 | 171 | 1 | 5693 | 3.00e-02 |
| 72 | [GO:0006956](http://www.ebi.ac.uk/ego/DisplayGoTerm?id=GO:0006956) | [complement activation](http://www.ebi.ac.uk/ego/DisplayGoTerm?id=GO:0006956) | 1 | 171 | 1 | 5693 | 3.00e-02 |
| 73 | [GO:0010831](http://www.ebi.ac.uk/ego/DisplayGoTerm?id=GO:0010831) | [positive regulation of myotube differentiation](http://www.ebi.ac.uk/ego/DisplayGoTerm?id=GO:0010831) | 1 | 171 | 1 | 5693 | 3.00e-02 |
| 74 | [GO:0070543](http://www.ebi.ac.uk/ego/DisplayGoTerm?id=GO:0070543) | [response to linoleic acid](http://www.ebi.ac.uk/ego/DisplayGoTerm?id=GO:0070543) | 1 | 171 | 1 | 5693 | 3.00e-02 |
| 75 | [GO:0030042](http://www.ebi.ac.uk/ego/DisplayGoTerm?id=GO:0030042) | [actin filament depolymerization](http://www.ebi.ac.uk/ego/DisplayGoTerm?id=GO:0030042) | 1 | 171 | 1 | 5693 | 3.00e-02 |
| 76 | [GO:0035729](http://www.ebi.ac.uk/ego/DisplayGoTerm?id=GO:0035729) | [cellular response to hepatocyte growth factor stimulus](http://www.ebi.ac.uk/ego/DisplayGoTerm?id=GO:0035729) | 1 | 171 | 1 | 5693 | 3.00e-02 |
| 77 | [GO:0051014](http://www.ebi.ac.uk/ego/DisplayGoTerm?id=GO:0051014) | [actin filament severing](http://www.ebi.ac.uk/ego/DisplayGoTerm?id=GO:0051014) | 1 | 171 | 1 | 5693 | 3.00e-02 |
| 78 | [GO:0051125](http://www.ebi.ac.uk/ego/DisplayGoTerm?id=GO:0051125) | [regulation of actin nucleation](http://www.ebi.ac.uk/ego/DisplayGoTerm?id=GO:0051125) | 1 | 171 | 1 | 5693 | 3.00e-02 |
| 79 | [GO:0060327](http://www.ebi.ac.uk/ego/DisplayGoTerm?id=GO:0060327) | [cytoplasmic actin-based contraction involved in cell motility](http://www.ebi.ac.uk/ego/DisplayGoTerm?id=GO:0060327) | 1 | 171 | 1 | 5693 | 3.00e-02 |
| 80 | [GO:0061041](http://www.ebi.ac.uk/ego/DisplayGoTerm?id=GO:0061041) | [regulation of wound healing](http://www.ebi.ac.uk/ego/DisplayGoTerm?id=GO:0061041) | 1 | 171 | 1 | 5693 | 3.00e-02 |
| 81 | [GO:0071364](http://www.ebi.ac.uk/ego/DisplayGoTerm?id=GO:0071364) | [cellular response to epidermal growth factor stimulus](http://www.ebi.ac.uk/ego/DisplayGoTerm?id=GO:0071364) | 1 | 171 | 1 | 5693 | 3.00e-02 |
| 82 | [GO:2000392](http://www.ebi.ac.uk/ego/DisplayGoTerm?id=GO:2000392) | [regulation of lamellipodium morphogenesis](http://www.ebi.ac.uk/ego/DisplayGoTerm?id=GO:2000392) | 1 | 171 | 1 | 5693 | 3.00e-02 |
| 83 | [GO:0030220](http://www.ebi.ac.uk/ego/DisplayGoTerm?id=GO:0030220) | [platelet formation](http://www.ebi.ac.uk/ego/DisplayGoTerm?id=GO:0030220) | 1 | 171 | 1 | 5693 | 3.00e-02 |
| 84 | [GO:0043534](http://www.ebi.ac.uk/ego/DisplayGoTerm?id=GO:0043534) | [blood vessel endothelial cell migration](http://www.ebi.ac.uk/ego/DisplayGoTerm?id=GO:0043534) | 1 | 171 | 1 | 5693 | 3.00e-02 |
| 85 | [GO:0002474](http://www.ebi.ac.uk/ego/DisplayGoTerm?id=GO:0002474) | [antigen processing and presentation of peptide antigen via MHC class I](http://www.ebi.ac.uk/ego/DisplayGoTerm?id=GO:0002474) | 1 | 171 | 1 | 5693 | 3.00e-02 |
| 86 | [GO:0002481](http://www.ebi.ac.uk/ego/DisplayGoTerm?id=GO:0002481) | [antigen processing and presentation of exogenous protein antigen via MHC class Ib, TAP-dependent](http://www.ebi.ac.uk/ego/DisplayGoTerm?id=GO:0002481) | 1 | 171 | 1 | 5693 | 3.00e-02 |
| 87 | [GO:0006003](http://www.ebi.ac.uk/ego/DisplayGoTerm?id=GO:0006003) | [fructose 2,6-bisphosphate metabolic process](http://www.ebi.ac.uk/ego/DisplayGoTerm?id=GO:0006003) | 1 | 171 | 1 | 5693 | 3.00e-02 |
| 88 | [GO:0002479](http://www.ebi.ac.uk/ego/DisplayGoTerm?id=GO:0002479) | [antigen processing and presentation of exogenous peptide antigen via MHC class I, TAP-dependent](http://www.ebi.ac.uk/ego/DisplayGoTerm?id=GO:0002479) | 1 | 171 | 1 | 5693 | 3.00e-02 |
| 89 | [GO:0050823](http://www.ebi.ac.uk/ego/DisplayGoTerm?id=GO:0050823) | [peptide antigen stabilization](http://www.ebi.ac.uk/ego/DisplayGoTerm?id=GO:0050823) | 1 | 171 | 1 | 5693 | 3.00e-02 |
| 90 | [GO:0042107](http://www.ebi.ac.uk/ego/DisplayGoTerm?id=GO:0042107) | [cytokine metabolic process](http://www.ebi.ac.uk/ego/DisplayGoTerm?id=GO:0042107) | 1 | 171 | 1 | 5693 | 3.00e-02 |
| 91 | [GO:0051291](http://www.ebi.ac.uk/ego/DisplayGoTerm?id=GO:0051291) | [protein heterooligomerization](http://www.ebi.ac.uk/ego/DisplayGoTerm?id=GO:0051291) | 1 | 171 | 1 | 5693 | 3.00e-02 |
| 92 | [GO:0043303](http://www.ebi.ac.uk/ego/DisplayGoTerm?id=GO:0043303) | [mast cell degranulation](http://www.ebi.ac.uk/ego/DisplayGoTerm?id=GO:0043303) | 1 | 171 | 1 | 5693 | 3.00e-02 |
| 93 | [GO:0002779](http://www.ebi.ac.uk/ego/DisplayGoTerm?id=GO:0002779) | [antibacterial peptide secretion](http://www.ebi.ac.uk/ego/DisplayGoTerm?id=GO:0002779) | 1 | 171 | 1 | 5693 | 3.00e-02 |
| 94 | [GO:0035279](http://www.ebi.ac.uk/ego/DisplayGoTerm?id=GO:0035279) | [mRNA cleavage involved in gene silencing by miRNA](http://www.ebi.ac.uk/ego/DisplayGoTerm?id=GO:0035279) | 1 | 171 | 1 | 5693 | 3.00e-02 |
| 95 | [GO:0001776](http://www.ebi.ac.uk/ego/DisplayGoTerm?id=GO:0001776) | [leukocyte homeostasis](http://www.ebi.ac.uk/ego/DisplayGoTerm?id=GO:0001776) | 1 | 171 | 1 | 5693 | 3.00e-02 |
| 96 | [GO:0001783](http://www.ebi.ac.uk/ego/DisplayGoTerm?id=GO:0001783) | [B cell apoptosis](http://www.ebi.ac.uk/ego/DisplayGoTerm?id=GO:0001783) | 1 | 171 | 1 | 5693 | 3.00e-02 |
| 97 | [GO:0002352](http://www.ebi.ac.uk/ego/DisplayGoTerm?id=GO:0002352) | [B cell negative selection](http://www.ebi.ac.uk/ego/DisplayGoTerm?id=GO:0002352) | 1 | 171 | 1 | 5693 | 3.00e-02 |
| 98 | [GO:0008635](http://www.ebi.ac.uk/ego/DisplayGoTerm?id=GO:0008635) | [activation of caspase activity by cytochrome c](http://www.ebi.ac.uk/ego/DisplayGoTerm?id=GO:0008635) | 1 | 171 | 1 | 5693 | 3.00e-02 |
| Downregulated transcripts | | | | | | | |
| 1 | [GO:0007155](http://www.ebi.ac.uk/ego/DisplayGoTerm?id=GO:0007155) | [cell adhesion](http://www.ebi.ac.uk/ego/DisplayGoTerm?id=GO:0007155) | 20 | 179 | 203 | 5693 | 4.58e-06 |
| 2 | [GO:0007165](http://www.ebi.ac.uk/ego/DisplayGoTerm?id=GO:0007165) | [signal transduction](http://www.ebi.ac.uk/ego/DisplayGoTerm?id=GO:0007165) | 13 | 179 | 240 | 5693 | 3.83e-02 |
| 3 | [GO:0008152](http://www.ebi.ac.uk/ego/DisplayGoTerm?id=GO:0008152) | [metabolic process](http://www.ebi.ac.uk/ego/DisplayGoTerm?id=GO:0008152) | 10 | 179 | 120 | 5693 | 4.28e-03 |
| 4 | [GO:0001568](http://www.ebi.ac.uk/ego/DisplayGoTerm?id=GO:0001568) | [blood vessel development](http://www.ebi.ac.uk/ego/DisplayGoTerm?id=GO:0001568) | 6 | 179 | 18 | 5693 | 1.20e-05 |
| 5 | [GO:0007507](http://www.ebi.ac.uk/ego/DisplayGoTerm?id=GO:0007507) | [heart development](http://www.ebi.ac.uk/ego/DisplayGoTerm?id=GO:0007507) | 6 | 179 | 52 | 5693 | 5.44e-03 |
| 6 | [GO:0016477](http://www.ebi.ac.uk/ego/DisplayGoTerm?id=GO:0016477) | [cell migration](http://www.ebi.ac.uk/ego/DisplayGoTerm?id=GO:0016477) | 5 | 179 | 21 | 5693 | 3.92e-04 |
| 7 | [GO:0007420](http://www.ebi.ac.uk/ego/DisplayGoTerm?id=GO:0007420) | [brain development](http://www.ebi.ac.uk/ego/DisplayGoTerm?id=GO:0007420) | 4 | 179 | 36 | 5693 | 2.54e-02 |
| 8 | [GO:0001569](http://www.ebi.ac.uk/ego/DisplayGoTerm?id=GO:0001569) | [patterning of blood vessels](http://www.ebi.ac.uk/ego/DisplayGoTerm?id=GO:0001569) | 4 | 179 | 21 | 5693 | 3.72e-03 |
| 9 | [GO:0001501](http://www.ebi.ac.uk/ego/DisplayGoTerm?id=GO:0001501) | [skeletal system development](http://www.ebi.ac.uk/ego/DisplayGoTerm?id=GO:0001501) | 4 | 179 | 23 | 5693 | 5.24e-03 |
| 10 | [GO:0060070](http://www.ebi.ac.uk/ego/DisplayGoTerm?id=GO:0060070) | [canonical Wnt receptor signaling pathway](http://www.ebi.ac.uk/ego/DisplayGoTerm?id=GO:0060070) | 3 | 179 | 19 | 5693 | 2.04e-02 |
| 11 | [GO:0007229](http://www.ebi.ac.uk/ego/DisplayGoTerm?id=GO:0007229) | [integrin-mediated signaling pathway](http://www.ebi.ac.uk/ego/DisplayGoTerm?id=GO:0007229) | 3 | 179 | 22 | 5693 | 3.03e-02 |
| 12 | [GO:0030890](http://www.ebi.ac.uk/ego/DisplayGoTerm?id=GO:0030890) | [positive regulation of B cell proliferation](http://www.ebi.ac.uk/ego/DisplayGoTerm?id=GO:0030890) | 3 | 179 | 13 | 5693 | 6.93e-03 |
| 13 | [GO:0030198](http://www.ebi.ac.uk/ego/DisplayGoTerm?id=GO:0030198) | [extracellular matrix organization](http://www.ebi.ac.uk/ego/DisplayGoTerm?id=GO:0030198) | 3 | 179 | 21 | 5693 | 2.68e-02 |
| 14 | [GO:0021915](http://www.ebi.ac.uk/ego/DisplayGoTerm?id=GO:0021915) | [neural tube development](http://www.ebi.ac.uk/ego/DisplayGoTerm?id=GO:0021915) | 3 | 179 | 11 | 5693 | 4.19e-03 |
| 15 | [GO:0050680](http://www.ebi.ac.uk/ego/DisplayGoTerm?id=GO:0050680) | [negative regulation of epithelial cell proliferation](http://www.ebi.ac.uk/ego/DisplayGoTerm?id=GO:0050680) | 3 | 179 | 15 | 5693 | 1.05e-02 |
| 16 | [GO:0030199](http://www.ebi.ac.uk/ego/DisplayGoTerm?id=GO:0030199) | [collagen fibril organization](http://www.ebi.ac.uk/ego/DisplayGoTerm?id=GO:0030199) | 3 | 179 | 12 | 5693 | 5.45e-03 |
| 17 | [GO:0001889](http://www.ebi.ac.uk/ego/DisplayGoTerm?id=GO:0001889) | [liver development](http://www.ebi.ac.uk/ego/DisplayGoTerm?id=GO:0001889) | 3 | 179 | 21 | 5693 | 2.68e-02 |
| 18 | [GO:0042742](http://www.ebi.ac.uk/ego/DisplayGoTerm?id=GO:0042742) | [defense response to bacterium](http://www.ebi.ac.uk/ego/DisplayGoTerm?id=GO:0042742) | 3 | 179 | 25 | 5693 | 4.23e-02 |
| 19 | [GO:0050731](http://www.ebi.ac.uk/ego/DisplayGoTerm?id=GO:0050731) | [positive regulation of peptidyl-tyrosine phosphorylation](http://www.ebi.ac.uk/ego/DisplayGoTerm?id=GO:0050731) | 3 | 179 | 19 | 5693 | 2.04e-02 |
| 20 | [GO:0014032](http://www.ebi.ac.uk/ego/DisplayGoTerm?id=GO:0014032) | [neural crest cell development](http://www.ebi.ac.uk/ego/DisplayGoTerm?id=GO:0014032) | 2 | 179 | 4 | 5693 | 5.66e-03 |
| 21 | [GO:0042310](http://www.ebi.ac.uk/ego/DisplayGoTerm?id=GO:0042310) | [vasoconstriction](http://www.ebi.ac.uk/ego/DisplayGoTerm?id=GO:0042310) | 2 | 179 | 3 | 5693 | 2.89e-03 |
| 22 | [GO:0006936](http://www.ebi.ac.uk/ego/DisplayGoTerm?id=GO:0006936) | [muscle contraction](http://www.ebi.ac.uk/ego/DisplayGoTerm?id=GO:0006936) | 2 | 179 | 5 | 5693 | 9.24e-03 |
| 23 | [GO:0050930](http://www.ebi.ac.uk/ego/DisplayGoTerm?id=GO:0050930) | [induction of positive chemotaxis](http://www.ebi.ac.uk/ego/DisplayGoTerm?id=GO:0050930) | 2 | 179 | 6 | 5693 | 1.36e-02 |
| 24 | [GO:0035019](http://www.ebi.ac.uk/ego/DisplayGoTerm?id=GO:0035019) | [somatic stem cell maintenance](http://www.ebi.ac.uk/ego/DisplayGoTerm?id=GO:0035019) | 2 | 179 | 7 | 5693 | 1.86e-02 |
| 25 | [GO:0045880](http://www.ebi.ac.uk/ego/DisplayGoTerm?id=GO:0045880) | [positive regulation of smoothened signaling pathway](http://www.ebi.ac.uk/ego/DisplayGoTerm?id=GO:0045880) | 2 | 179 | 6 | 5693 | 1.36e-02 |
