# Supplementary material for: Transcriptome analysis of chicken kidney tissues following coronavirus avian infectious bronchitis virus infection
Source: BMC Genomics. 2013 Oct 30;14:743. doi: 10.1186/1471-2164-14-743 (PMC3870970; doi:10.1186/1471-2164-14-743)
Supplement: Additional file 2 — The p -value of KEGG pathway analysis. [file 1471-2164-14-743-S2.doc]

**Additional file 2**

The *p*-value of KEGG pathway analysis

| **KEGG pathway** | **p-value** |
| --- | --- |
| Focal adhesion | 4.68e-04 |
| Cytokine-cytokine receptor interaction | 4.89e-03 |
| Cell adhesion molecules (CAMs) | 1.4e-03 |
| ECM-receptor interaction | 4.91e-03 |
| PPAR signaling pathway | 1e-04 |
| Peroxisome | 1.45e-03 |
| Gap junction | 4.31e-02 |
| Adherens junction | 3.09e-02 |
| Valine, leucine and isoleucine degradation | 7.7e-03 |
| Citrate cycle (TCA cycle) | 3.27e-04 |
| Tryptophan metabolism | 1.62e-02 |
| Drug metabolism - cytochrome P450 | 3.03e-03 |
